# The Bicycle and the Perineum

**Published:** 1897-02

**Authors:** 


ARTICLE VIII.

### THE BICYCLE AND THE PERINEUM.

An experience of several years with the bicycle has convinced us that its future usefulness is bound to be greatly restricted, especially for elderly men, unless a form of saddle can be devised which will not press upon the perineum as does the present saddle. Of late, considerable attention has been given to disturbances of the bladder, urethra and prostrate which have been recognized as due to this pressure. Although the greatest ingenuity has been expended in improving the wheel in all its other parts, the saddle is practically the same as it was ten years ago. Many men who have learned to ride the wheel have found so much discomfort following its use that they have felt obliged to give up a form of exercise, which, to our minds, is the most healthful, the most exhilarating and the most pleasurable of all out-door sports, equally enjoyed by both sexes and all ages, from infancy to old age. The latest acquisition to the ranks of wheelmen is an octogenarian.

Mr. Arthur Chas. Roper, F. R. C. S., etc., an English surgeon and a bicycle enthusiast, has written in *The Lancet*, for May, the very best paper on the subject of bicycle seats that it has been our fortune to read. He gives a most interesting account of his personal experience with his wheel during last summer. He says that when he rode any considerable distance, and at a good pace, he found it necessary to stop and urinate every hour, or oftener. He found that his penis was cold and shrunken, the scrotum was also in a similar condition. The return to the normal state was attended with considerable aching. When urinating, the last drops were not shut off promptly. He also found that he had to urinate oftener at night. He says the many bicyclists have complained to him of the same symptoms. At times he suffered so greatly from pubic pains and frequent micturition that he was forced to seek relief in hot baths and opiates. "I was soon forced," he says, "to the unpleasant diagnosis of cystitis, which I could not and can not doubt was traumatic in its origin, and was probably started by bruising and constant jarring of the balbous portion of the uretha." In order to show how these disturbances may be brought about, he gives a brief but concise description of the anatomy of the perineum. "Between the skin and the bone the inferior pudendal nerve, and between the skin and triangular ligament the bulb, urethra and superficial perineal nerves, and the accelerator, urinæ, and erector penis muscles with their nerves." Pressure exerted between the sides of the pubic arch must compress these structures more or less against the bone and the unyielding triangular ligament, thus the pressure on the sensory nerves of the part, and on the motor nerves which supply the muscles of the urethra, accounts for the shrivelling and aching of the penis and inability to completely empty the urethra after micturition.

The writer then demonstrates that the ordinary saddle fulfills the conditions for producing just the pressure to cause all this trouble. In discussing a form of saddle which will relieve the perineum of this pressure, he says that as 'the transverse diameter of the outlet of the pelvis is four inches, and one and one half inches must be added to either side, if we are to derive full benefit from our natural padding, there must be at least seven inches of suspended leather behind upon which to sit. Even with a saddle thus constructed, the moment we begin to pedal, our muscles, acting upon the saddle as a fulcrum, force us forward to its narrow portion. If we attempt to overcome this by tilting the saddle, the peak at once presses against the perineum. The same thing happens when the rider leans forward, as most persons do when riding against the wind." Our anthor sums np the whole matter of saddles and seats by saying that "in spite of the advertisements of the makers of anatomical seats and the opinions of many eminent medical men, I contend that, for the work which has to be done on it, the saddle type of seat is the best, though I contend, with an equal conviction, that the ordinary make of saddle is bad and deleterious." As the result of a personal study of upwards of eighty seats and saddles, he finds none which are entirely free from objections. Those without points, that is seats, which do not press upon the perineum, are not easy to ride, as the rider is not secure from falls liable to follow sudden jars.

Dr. Roper recommends the *seat* for those having prostatic trouble, and who do not care to ride fast. These points should be carefully noted by physicians, for many obscure troubles of the bladder and adjacent parts may be due to the pressure of the saddle. Elderly men especially should be warned of possible danger from excessive riding of the bicycle, as saddles are now constructed. A great field is open to the inventor in studying a form of saddle which will be at the same time easy to ride and free from danger.—*Pacific Medical Journal.*

---

